# Scoparone alleviates Ang II‐induced pathological myocardial hypertrophy in mice by inhibiting oxidative stress

**DOI:** 10.1111/jcmm.16304

**Published:** 2021-02-09

**Authors:** Linmao Lyu, Jiazheng Chen, Wei Wang, Tao Yan, Jiamao Lin, Hongmei Gao, Hui Li, Ruijuan Lv, Feng Xu, Lijun Fang, Yuguo Chen

**Affiliations:** ^1^ Department of Emergency Medicine Qilu Hospital of Shandong University Jinan China; ^2^ Shandong Provincial Clinical Research Center for Emergency and Critical Care Medicine, Institute of Emergency and Critical Care Medicine of Shandong University, Chest Pain Center, Qilu Hospital of Shandong University Jinan China; ^3^ Key Laboratory of Emergency and Critical Care Medicine of Shandong Province, Key Laboratory of Cardiopulmonary‐Cerebral Resuscitation Research of Shandong Province, Shandong Provincial Engineering Laboratory for Emergency and Critical Care Medicine, Qilu Hospital of Shandong University Jinan China; ^4^ The Key Laboratory of Cardiovascular Remodeling and Function Research, Chinese Ministry of Education, Chinese Ministry of Health and Chinese Academy of Medical Sciences: The State and Shandong Province Joint Key Laboratory of Translational Cardiovascular Medicine; The State and Shandong Province Joint Key Laboratory of Translational Cardiovascular Medicine; Qilu Hospital of Shandong University Jinan China; ^5^ Department of Joint Surgery Shandong Provincial Hospital Cheeloo College of Medicine Shandong University Jinan China; ^6^ School of Public Health Shandong University Jinan China; ^7^ Department of Thoracic Surgery Shandong Provincial Hospital Cheeloo College of Medicine Shandong University Jinan China; ^8^ Department of Internal Medicine‐Oncology Shandong Cancer Hospital and Institute Shandong First Medical University and Shandong Academy of Medical Sciences Jinan China; ^9^ Department of Cardiology The Second Affiliated Hospital of Shandong University of Traditional Chinese Medicine Jinan China; ^10^ Department of Emergency Medicine The Affiliated Hospital of Shandong University of Traditional Chinese Medicine Jinan China; ^11^ Department of Traditional Chinese Medicine Shandong Academy of Occupational Health and Occupational Medicine Shandong First Medical University and Shandong Academy of Medical Sciences Jinan China

**Keywords:** angiotensin II, fibrosis, hypertrophy, oxidative stress, scoparone

## Abstract

Long‐term poorly controlled myocardial hypertrophy often leads to heart failure and sudden death. Activation of ras‐related C3 botulinum toxin substrate 1 (RAC1) by angiotensin II (Ang II) plays a pivotal role in myocardial hypertrophy. Previous studies have demonstrated that scoparone (SCO) has beneficial effects on hypertension and extracellular matrix remodelling. However, the function of SCO on Ang II‐mediated myocardial hypertrophy remains unknown. In our study, a mouse model of myocardial hypertrophy was established by Ang II infusion (2 mg/kg/day) for 4 weeks, and SCO (60 mg/kg bodyweight) was administered by gavage daily. In vitro experiments were also performed. Our results showed that SCO could alleviate Ang II infusion‐induced cardiac hypertrophy and fibrosis in mice. In vitro, SCO treatment blocks Ang II‐induced cardiomyocyte hypertrophy, cardiac fibroblast collagen synthesis and differentiation to myofibroblasts. Meanwhile, we found that SCO treatment blocked Ang II‐induced oxidative stress in cardiomyocytes and cardiac fibroblasts by inhibiting RAC1‐GTP and total RAC1 in vivo and in vitro. Furthermore, reactive oxygen species (ROS) burst by overexpression of RAC1 completely abolished SCO‐mediated protection in cardiomyocytes and cardiac fibroblasts in vitro. In conclusion, SCO, an antioxidant, may attenuate Ang II‐induced myocardial hypertrophy by suppressing of RAC1 mediated oxidative stress.

## 1 INTRODUCTION

Pathological myocardial hypertrophy is the common feature of various cardiovascular diseases, such as hypertension, valve disease, myocardial infarction and congenital heart disease. It is characterized by cardiac hypertrophy and interstitial fibrosis. Long‐term poorly controlled myocardial hypertrophy often leads to heart failure and sudden death in patients with the above‐mentioned diseases.^1^, ^2^

The mechanism of myocardial hypertrophy is very complicated; however, angiotensin II (Ang II) has been demonstrated to play a pivotal role in the pathogenesis.^3^, ^4^ Ras‐related C3 botulinum toxin substrate 1 (RAC1) is a member of the Rho family of GTPases, that cycles between active (GTP‐bound) and inactive (GDP‐bound) states.^5^ Activation of RAC1 is required for Ang II‐induced cardiac hypertrophy, fibrosis and intracellular oxidation in adult hearts.^6^, ^7^, ^8^, ^9^ In the cardiovascular system, RAC1 is a major regulator of NADPH oxidase (NOX) activity. In cardiac fibroblasts and cardiomyocytes, Ang II can activate RAC1 (increasing expression of RAC1‐GTP) and NADPH oxidase (NOX).^7^, ^10^, ^11^ NOX is a family of seven enzymes that participate in generating O^2−^ by catalysing the electron transfer from NADPH to O^2^. Among the NOX family, NOX2 and NOX4 are mainly expressed in cardiomyocytes and cardiac fibroblasts^12^ and are involved in cardiac hypertrophy and fibrosis.^13^, ^14^

Scoparone (6,7‐dimethoxycoumarin, SCO) is one of the primary active ingredients isolated from the shoot of *Artemisia* capillaries. In the cardiovascular system, SCO was reported to be a potent hypotensive drug, with a probable mechanism of vascular dilatory action based on calcium mobilization regulation.^15^ SCO could inhibit Ang II‐induced extracellular matrix remodelling in cardiac fibroblasts in vitro at least partly by inhibiting TGF‐β1/Smad signalling.^16^ SCO also showed a protective effect against ischaemia‑reperfusion‑induced myocardial injury by counteracting oxidative stress.^17^ However, the effect of SCO on Ang II‐induced myocardial hypertrophy is still unknown.

In this study, we discovered that SCO down‐regulated Ang II‐induced activation of RAC1 and oxidative stress both in cardiomyocytes and cardiac fibroblasts. We also demonstrate that SCO‐mediated inhibition of RAC1 alleviates Ang II‐induced cardiac hypertrophy and fibrosis in vitro and in vivo. To our knowledge, this is the first report to describe SCO as an inhibitor of RAC1 and agent of anti‐myocardial hypertrophy.

## 2 MATERIALS AND METHODS

### 2.1 Materials

Scoparone (E‐0358) was purchased from Tauto (Shanghai, China). Ang II (A9525) was purchased from Sigma‐Aldrich (St. Louis, MO, USA), Alzet osmotic minipump (model 2004) was purchased from DURECT (California, USA), tail‐cuff instrument was purchased from IITC Inc (Woodland Hills, California, USA), and the ultrasound imaging system(Vevo2100) was purchased from VisualSonics (Toronto, Canada). Antibodies against 4HNE (ab46545), RAC1‐GTP (ab203884), α‐actinin (ab9465), vimentin (ab8978), collagen I (ab34710), collagen III (ab7778) and connective tissue growth factor (CTGF, ab6992) were purchased from Abcam (Cambridge, MA, USA). Antibody against α‐smooth muscle actin (α‐SMA, A2547, for Western blot use) was purchased from Sigma‐Aldrich (St. Louis, MO, USA). Antibodies against total‐RAC1 (66122‐1‐lg), NOX2 (19013‐1‐AP), NOX4 (14347‐1‐AP), α‐SMA (14395‐1‐AP, for immunofluorescence stain use), cardiac troponin I (CTNI, 66376‐1‐Ig) and β‐actin (60008‐1‐Ig) were purchased from Proteintech (Hubei, China). ^3^H‐leucine (NET135H) was purchased from Perkin Elmer (Waltham, MA, USA), ROS assay (S0033) was purchased from Beyotime (Shanghai, China), and the Cytotoxicity Detection Kit (11644793001) was purchased from Roche (Hoffmann, Germany).

### 2.2 Animals and treatments

C57BL/6 mice (male, 8 weeks old) were purchased from HUAFUKANG Bioscience Co. (Beijing, China). The animals were housed in an air‐conditioned room (temperature 20 ± 1℃, humidity 60 ± 10%, a light cycle of 12 hours). All mice had free access to food and water. All mouse experiments in this study were performed in accordance with the National Institutes of Health (NIH) Guide for the Care and Use of Laboratory Animals. The protocol was approved by the Committee on the Ethics of Animal Experiments of Shandong First Medical University and Shandong Academy of Medical Sciences.

A mouse model of myocardial hypertrophy was established by Ang II infusion for 4 weeks adapted from previous reports.^4^, ^18^ SCO was mixed in a 0.5% carboxyl methyl cellulose (CMC) solution. Briefly, 29 mice at age of 8 weeks were randomly divided into four groups: (a) control group mice(n = 7) were treated with the subcutaneous delivery of vehicle saline(0.9% NaCl) via Alzet osmotic minipumps (model 2004) with an infusion rate of 0.25 µL/h and gavage administration of the CMC solution; (b) Ang II group mice (n = 7) were treated with subcutaneous delivery of Ang II (2 mg/kg/day) dissolved in saline via Alzet osmotic minipumps and gavage administration of the CMC solution; (C) Ang II + SCO group mice (n = 8) were treated with subcutaneous delivery of Ang II and gavage administration of SCO (60 mg/kg bodyweight) in CMC solution daily as previously reported^19^; and (d) SCO group mice (n = 7) were treated with the same amount of SCO and subcutaneous delivery of vehicle saline as described above. Blood pressure and bodyweight were measured every week.

### 2.3 Echocardiography and blood pressure

After 4‐week treatment, transthoracic echocardiography was performed in mice with a high‐frequency ultrasound imaging system, Vevo2100 equipped with a 30‐MHz imaging transducer. All recordings were acquired in animals under light anaesthesia with automated isoflurane inhalation and positioned on a plank in a supine position. Two‐dimensionally guided M‐mode images at the papillary muscle level were obtained from the parasternal long‐axis view. Echocardiographic parameters, including LVPWd (left ventricular posterior wall thickness end diastole), LVIDd (left ventricular internal diameter end diastole), LVIDs (left ventricular internal diameter end systole), IVSd (interventricular septum thickness end diastole), LVEDD (left ventricular end‐diastolic diameter) and LVESD (left ventricular end‐systolic diameter), were measured in mice from the four groups (n = 7‐8). LV fractional shortening (FS) was calculated as LVFS (%) = (LVEDD − LVESD)/LVEDD) × 100.

Systolic blood pressure (SBP) and diastolic blood pressure (DBP) were measured in awake mice non‐invasively by the tail‐cuff instrument. Mean arterial pressure (MAP) was calculated as MAP = (SBP + 2 × DBP)/3.

### 2.4 Histology and immunohistochemistry

Euthanized mice were perfused with saline to eliminate blood. Hearts were harvested, dried on gauze, weighed, dissected and fixed in 10% formalin or frozen in liquid nitrogen. After fixing for 24 hours, heart tissues were paraffin‐embedded and cut into 5 μm serial sections. Haematoxylin‐eosin (HE) staining was applied to show the morphology of cardiac ventricles. The cardiomyocyte cross‐sectional area (CSA) was visualized via wheat germ agglutinin (WGA) staining. To evaluate cardiac fibrosis, tissue sections were stained with a Masson's Trichrome Kit. In Masson's staining, collagen was dyed blue. 4‐Hydroxy‐2‐Nonenal (4‐HNE) is a by‐product of lipid peroxidation and is widely accepted as a stable marker of oxidative stress. The immunohistochemistry of 4‐HNE was performed as previously described.^20^ CSAs (μm^2^), fractional area of collagen content (%) and level of 4‐HNE (fold change) were analysed with software Image‐Pro Plus 6.0 (IPP 6.0, Media Cybernetics, USA).

### 2.5 Immunofluorescence stain

Immunofluorescence staining of heart sections and isolated cells was carried out according to manufacturer's instructions. To show α‐SMA expression of cardiac fibroblasts in heart tissues, the sections were incubated with a rabbit α‐SMA antibody combined with a mouse vimentin antibody. Then, the number of vimentin(+) cells and α‐SMA (+) cells was counted.

For staining the Rac‐1‐GTP of cardiomyocytes or cardiac fibroblasts in heart tissues, the sections were incubated with a rabbit RAC1‐GTP antibody combined with a mouse α‐actinin antibody or a mouse vimentin antibody. Fluorescent images were obtained using a fluorescent microscope. Calculation and analysis were based on six randomly selected fields from four hearts in each group. The fluorescence intensities of Rac‐1‐GTP in vimentin(+) and α‐actinin(+) cells (fibroblasts and cardiomyocytes, respectively) were quantified with IPP 6.0 and expressed as a relative value.

### 2.6 Measurement of malondialdehyde (MDA)

MDA is a marker of lipid peroxidation and oxidative stress. The level of MDA in the heart tissue was detected using a thiobarbituric acid (TBA)‐based assay kit. MDA can react with TBA to form a colorimetric product, proportional to the MDA present. The intensity of the colour was measured spectrophotometrically at 532 nm.^21^

### 2.7 Western blot

Total protein was extracted and quantified from cells and tissues using the method described previously.^3^ Equal amounts of protein were subjected to sodium dodecyl sulphate‐polyacrylamide gel electrophoresis (SDS‐PAGE) and were transferred onto 0.2 μm polyvinylidene fluoride (PVDF) membranes. Membranes were blocked with 5% skimmed milk solution and incubated with the primary and secondary antibodies. Specific protein expression levels were normalized to β‐actin protein levels in total cell lysates.

### 2.8 Reverse transcription polymerase chain reaction (RT‐PCR) and quantitative real‐time PCR (Q‐PCR)

Total RNAs from heart tissues and neonatal rat cardiomyocytes (NRCMs) were extracted using TRIzol reagent, and reverse transcription reactions were performed with 0.5 µg RNA using a First Strand cDNA Synthesis Kit. Expression levels of target genes were normalized by concurrent measurement of glyceraldehyde‐3‐phosphate dehydrogenase (GAPDH) mRNA levels. Primers used for Q‐PCR are summarized in Table S1.

### 2.9 Isolation and culture of neonatal rat cardiomyocytes and fibroblasts

NRCMs and neonatal rat fibroblasts (NRCFs) were isolated and cultured as previously described.^3^ The isolated cardiomyocytes were seeded onto gelatin‐coated plastic culture dishes at a density of 5 × 10^4^ cells/cm^2^ in low glucose (1 g/L) Dulbecco's modified Eagle's medium (DMEM) supplemented with 8% horse serum, 5% new‐born calf serum, penicillin (100 U/mL) and streptomycin (100 mg/mL). The isolated fibroblasts were maintained in low glucose (1 g/L) DMEM supplemented with 10% foetal bovine serum, penicillin (100 U/mL) and streptomycin (100 mg/mL). Cardiac fibroblasts at passage one were used in cell growth curve assay, and passage two or three cells were used for the other experiments.

### 2.10 ^3^H‐leucine incorporation

NRCMs seeded in 24‐well plates were treated with different stimuli and ^3^H‐leucine (final concentration 1 μCi/mL) as indicated for 48 hours. After being washed with ice‐cold phosphate‐buffered saline (PBS) two times, the cells were precipitated with ice‐cold 5% trichloroacetic acid (TCA) for 30 minutes and then washed with ice‐cold 5% TCA two times followed by two additional washes with ice‐cold PBS. Finally, the cells were lysed with 0.2 mL per well of 0.5 mol/L NaOH for 30 minutes at 37°C. The radioactivity of ^3^H‐leucine in cell lysates was measured with a scintillation counter.

### 2.11 Measure of reactive oxygen species (ROS)

Cells were cultured in 6‐well plates and treated for the indicated time and then were incubated with 10 μmol/L DCFH‐DA in serum‐free medium at 37°C for 30 minutes in the dark, washed with PBS and observed under a fluorescence microscope. For each experiment, 8 fields were chosen randomly to acquire pictures, and the integrated optical density (IOD) of the images was quantified with IPP 6.0.

### 2.12 LDH release assay

Death of NRCMs and NRCFs in vitro was spectrophotometrically measured by LDH release assay with Cytotoxicity Detection Kit, which was measured in optical density (OD). Briefly, cells were cultured in serum‐free DMEM with stimulations for 48 hours, and 100 μL of the supernatant was incubated with 100 μL reaction mixture (provided in the kit) at room temperature for 15 minutes in the dark. The absorbance of samples was measured with a microplate reader at a wavelength of 490 nm.

### 2.13 Statistical analysis

Data are presented as the mean ± SD. Statistical analyses were performed by one‐way ANOVA followed by the Student‐Newman‐Keuls test with SPSS10.0 (SPSS Inc, Chicago, IL, USA). Probability (*P*) values < 0.05 were considered statistically significant.

## 3 RESULTS

### 3.1 SCO attenuates Ang II‐induced pathological cardiac hypertrophy in vivo

**FIGURE 1:**
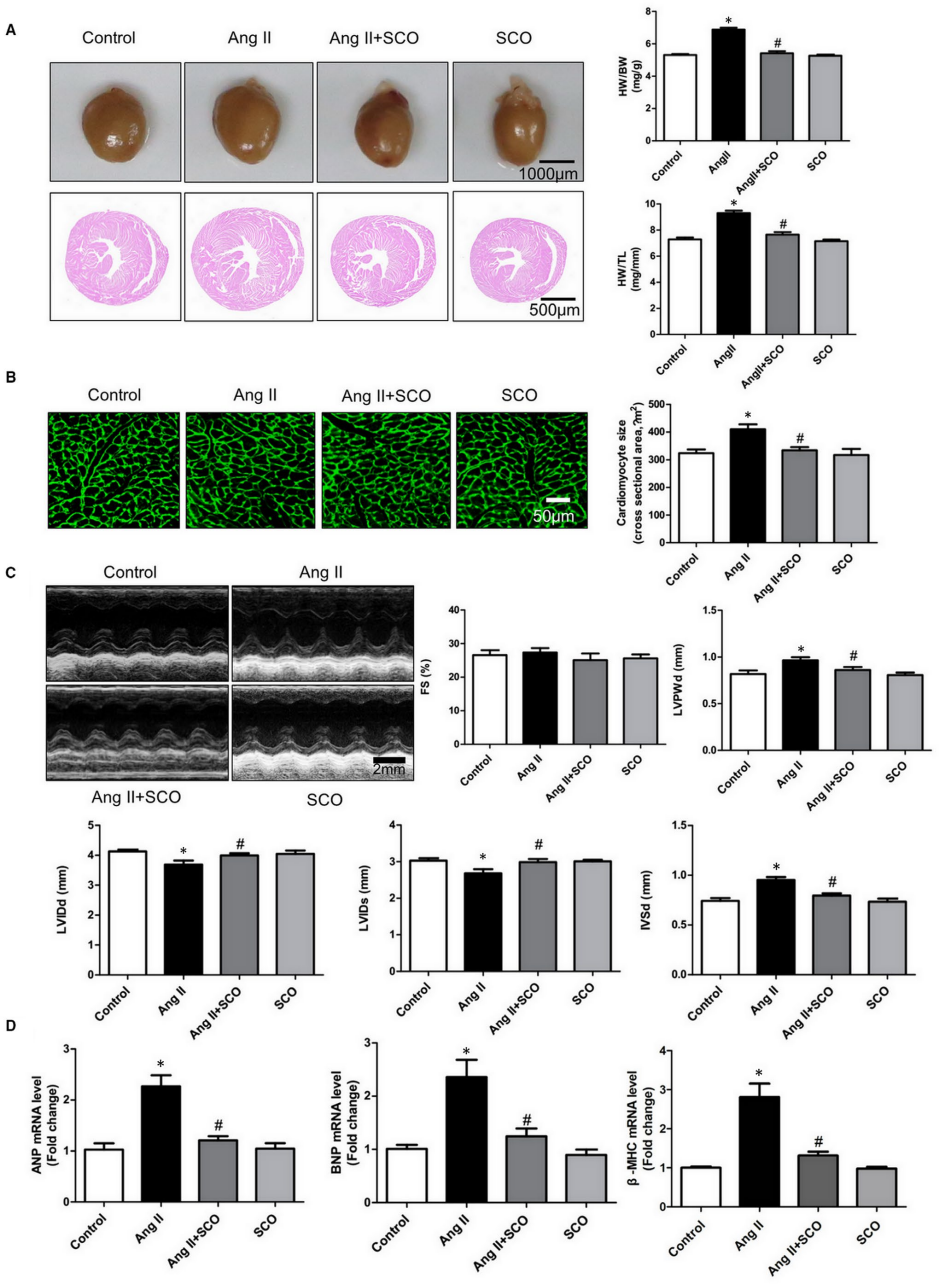
Scoparone inhibits Ang II infusion‐induced pathological cardiac hypertrophy in vivo. A, Heart weight /bodyweight ratio (HW/BW), heart weight/tibia length ratio (HW/TL) in mice from the four groups (n = 7 or 8 per group). B, Representative photographs of wheat germ agglutinin (WGA) stained sections and data of cardiomyocytes cross‐sectional area (CSA, n = 4).C, Echocardiographic parameters, including FS (fractional shortening), LVPWd (left ventricular posterior wall thickness end diastole), LVIDd (left ventricular internal diameter end diastole), LVIDs (left ventricular internal diameter end systole) and IVSd (interventricular septum thickness end diastole) were evaluated and calculated (n = 7 or 8). D, Real‐time PCR analysis of ANP (atrial natriuretic peptide), BNP (brain natriuretic peptide) and β‐myosin heavy chain (β‐MHC) mRNA level (n = 4).*P < 0.05 versus control group; ^#^ *P* < 0.05 versus Ang II group

Ang II infusion is a classic method to establish a myocardial hypertrophy model in mice. Long time Ang II infusion results in cardiac hypertrophy. As shown in Figure 1A,B, SCO treatment significantly reduced the heart weight /bodyweight ratio (HW/BW), heart weight/tibia length ratio (HW/TL) and cardiomyocyte cross section area (CSA) in mice subjected to Ang II infusion. Meanwhile, compared to the Ang II group, SCO treatment decreased LVPWd and IVSd and increased LVIDd and LVIDs (Figure 1C). In addition, Ang II infusion up‐regulated the levels of atrial natriuretic peptide (ANP), brain natriuretic peptide (BNP) and beta‐myosin heavy chain (β‐MHC) in the hearts of mice, and SCO treatment decreased these changes (Figure 1D). In addition, SCO significantly lowered Ang II‐elevated blood pressure (SBP, DBP and MAP) at different time‐points, but it was not able to normalize the blood pressure (Figure S1).

### 3.2 SCO attenuates Ang II‐induced cardiomyocytes hypertrophy in vitro

To further explore the anti‐hypertrophic effect of SCO, neonatal rat cardiomyocytes (NRCMs) were isolated and cultured, and Ang II was used to induce cardiomyocyte hypertrophy in vitro. In the LDH assay, 10‐200 μmol/L SCO showed no toxicity to NRCMs (Figure S2A). The ^3^H‐leucine incorporation rate reflects the efficiency of protein synthesis in cells and is a quantifiable index of cardiomyocyte hypertrophy in vitro.^22^, ^23^ Figure S2B,C shows that SCO attenuated Ang II‐induced cardiomyocyte ^3^H‐leucine incorporation and cell surface enlargement in a dose‐dependent manner. These results indicate that SCO can block Ang II‐induced cardiac hypertrophic response in vitro.

### 3.3 SCO attenuates Ang II‐induced cardiac fibrosis in vivo

**FIGURE 2:**
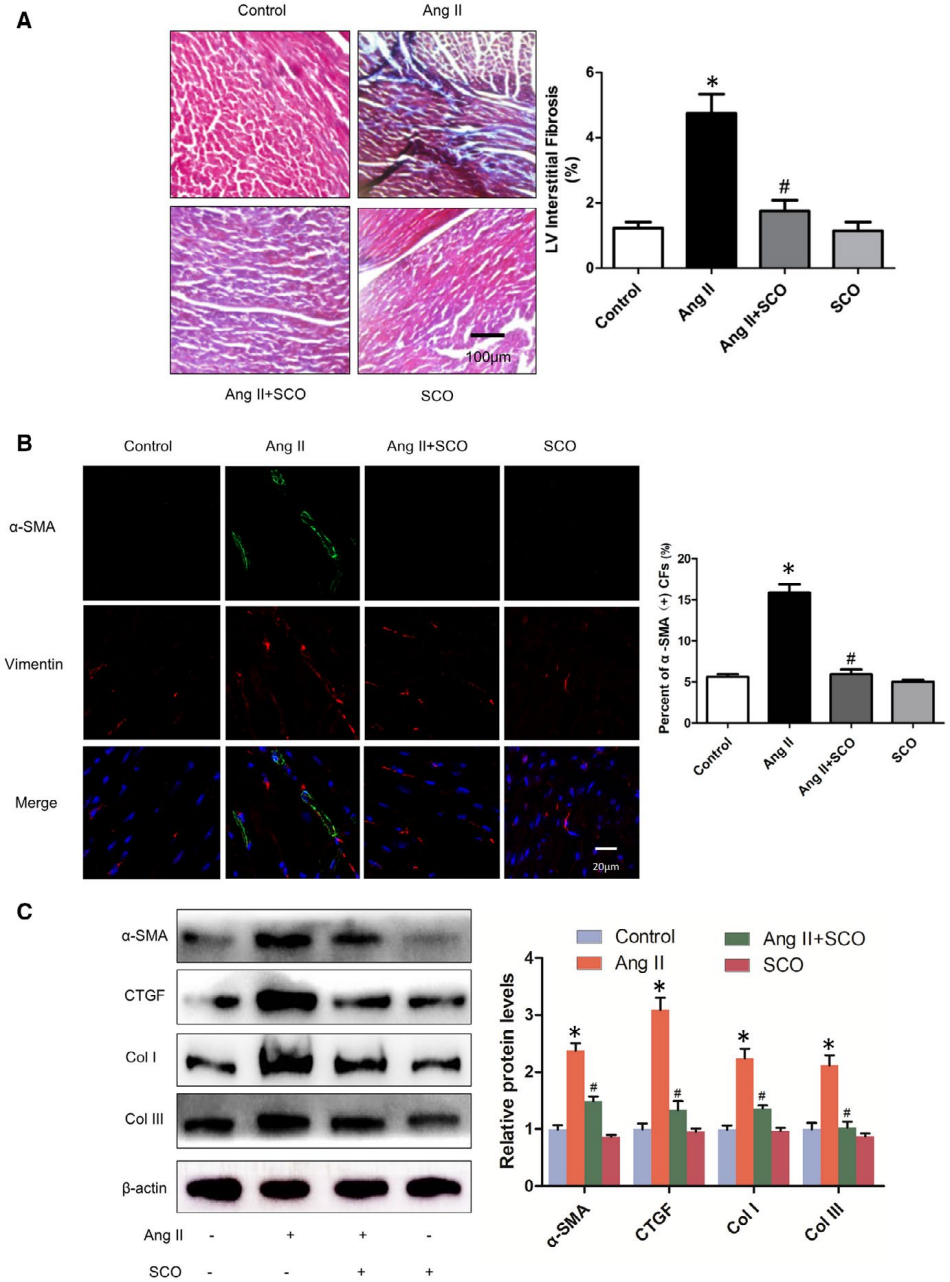
Scoparone inhibits Ang II infusion‐induced cardiac fibrosis in vivo. A, Masson's stain of left ventricle in mice from the four groups (n = 4). B, Immunofluorescence of α‐smooth muscle actin (α‐SMA, green) and vimentin (VIM, red, marker of fibroblasts), percentage of α‐SMA‐positive cardiac fibroblasts(CFs) were calculated (n = 4). C, level of fibrosis related proteins (n = 3).*P < 0.05 versus control group; ^#^ *P* < 0.05 versus Ang II group

In Ang II‐induced cardiac remodelling, cardiac hypertrophy and fibrosis are often accompanied. As shown in Figure 2A, Ang II + SCO‐treated mice exhibited a significant decrease in average collagen volume compared with Ang II group mice. To further confirm this finding, we detected the expression ofα‐SMA in the fibroblasts of mice hearts. Vimentin is a marker of fibroblasts, and high α‐SMA expression in vimentin (+) cells represents the transformation from fibroblasts to myofibroblasts. Myofibroblast is an activated form of fibroblast, which has a stronger pro‐fibrogenic ability. In Figure 2B, immunofluorescent staining of α‐SMA and vimentin showed that Ang II infusion significantly increased the population of α‐SMA and vimentin‐positive cells in mouse hearts, and SCO administration blocked the switch of fibroblasts to myofibroblasts in vivo. Accordingly, SCO treatment also dramatically reduced the protein levels of α‐SMA, CTGF, collagen I and collagen III in the hearts of Ang II‐infused mice (Figure 2C).

### 3.4 SCO attenuates Ang II‐induced cardiac fibroblasts differentiation and collagen synthesis in vitro

As Figure S3A showed, 10‐200 μmol/L SCO had no cytotoxicity to NRCFs. Next, we assessed the anti‐fibrotic effect of SCO on NRCFs. In Figure S3B,C, decreased expression of α‐SMA indicating that SCO suppressed Ang II‐induced NRCFs differentiation from fibroblasts to myofibroblasts in vitro. In agreement with these results, SCO significantly inhibited Ang II‐induced expression of CTGF, collagen I and collagen III in NRCFs (Figure S3C).

### 3.5 SCO alleviates Ang II‐induced oxidative stress of cardiomyocytes and cardiac fibroblasts in vivo

**FIGURE 3:**
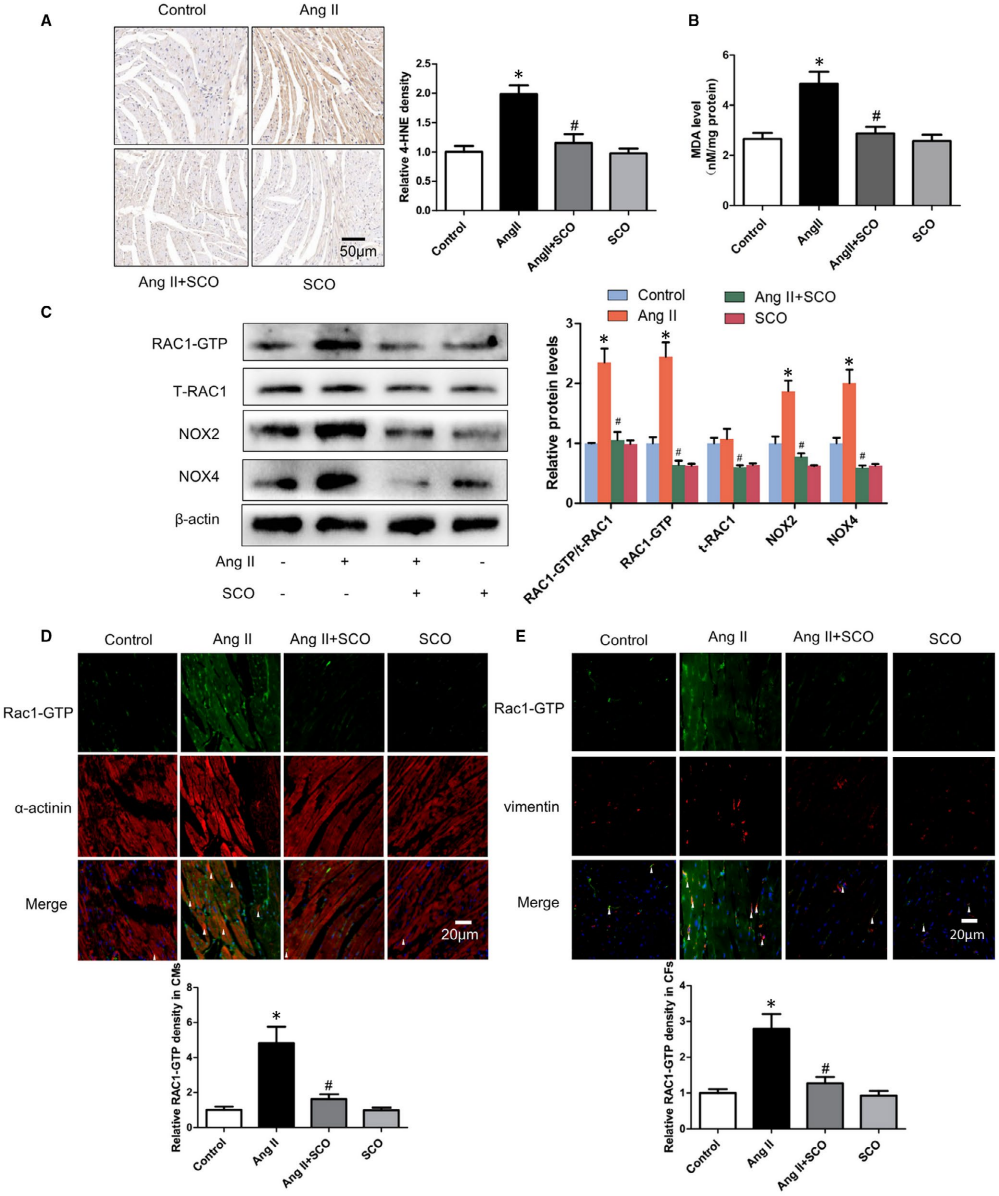
Scoparone inhibits Ang II infusion‐induced oxidative stress of hearts in vivo. A, 4‐HNE immunohistochemical staining of left ventricular (LV) (n = 4). B, MDA level of hearts, results are expressed as nmol/mg of protein (n = 7‐8). C, measure of RAC1‐GTP, total RAC1, NOX2 and NOX4 in hearts with Western blot (n = 3). D, Rac1‐GTP immunofluorescence staining in cardiomyocytes (CMs, α‐actinin positive) in vivo (n = 4). E, Rac1‐GTP immunofluorescence staining in cardiac fibroblasts (CFs, vimentin positive) in vivo (n = 4).*P < 0.05 vs control group; ^#^ *P* < 0.05 vs Ang II group

To investigate the anti‐oxidative effect of SCO in Ang II‐induced myocardial oxidative stress, immunohistochemical staining of 4‐HNE and measurement of MDA were performed. 4‐HNE and MDA are markers of lipid peroxidation and oxidative stress.^20^, ^24^ The results (Figure 3A,B) showed that Ang II leads to an increase in 4‐HNE and MDA levels in the hearts, whereas the effect was significantly suppressed by SCO treatment in the Ang II + SCO group. Meanwhile, as shown in Figure 3C, in response to the sustained Ang II stimulation, the levels of RAC1‐GTP, RAC1‐GTP/total‐RAC1 and downstream proteins of RAC1, including NOX2 and NOX4, were up‐regulated. SCO treatment suppressed the elevated levels of RAC1‐GTP, RAC1‐GTP/total‐RAC1, NOX2 and NOX4. The level of total RAC1 was not changed by Ang II infusion, but SCO administration inhibited the level of total RAC1 in mouse hearts.

To distinguish the expression of Rac1‐GTP in cardiomyocytes and cardiac fibroblasts in hearts, RAC1‐GTP was co‐immunostained separately with α‐actinin and vimentin. Figure 3D,E shows that SCO treatment alleviated Ang II‐induced RAC1‐GTP both in cardiomyocytes and cardiac fibroblasts in vivo.

### 3.6 Protein level of NOX2 and NOX4 could be regulated by RAC1 in cardiomyocytes and cardiac fibroblasts

Increasing RAC1‐GTP/total RAC1 ratio manifests activation of RAC1. As shown in Figure S4A,B, Ang II increased the levels of RAC1‐GTP/total RAC1, RAC1‐GTP, NOX2 and NOX4 in NRCMs and NRCFs in a concentration‐dependent manner. This is consistent with previous reports. The role of RAC1 in protein level regulation of NOX2 and NOX4 is still not clear. To explore the role of RAC1 in regulating the expression of NOX2 and NOX4, three doses of RAC1 overexpression adenovirus (1‐10 moi) were applied. The results showed that overexpression of RAC1 increased the protein levels of NOX2 and NOX4 in NRCMs and NRCFs (Figure S5A,B).

### 3.7 SCO inhibits Ang II‐induced cardiomyocytes hypertrophy by suppressing RAC1 mediated oxidative stress

**FIGURE 4:**
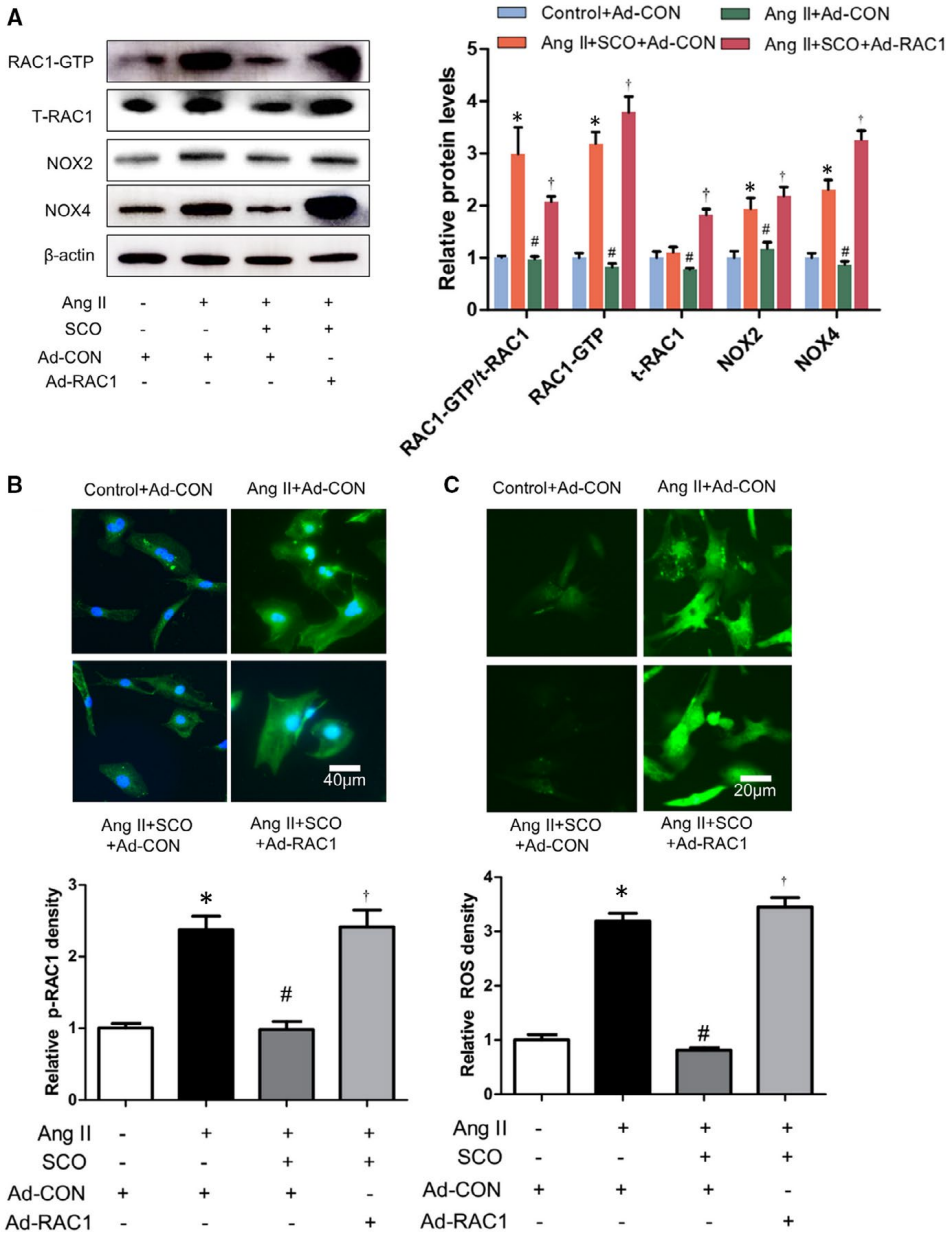
Scoparone inhibits Ang II‐induced cardiomyocytes oxidative stress by suppressing Rac1. NRCMs were treated with Ang II (1 μmol/L), scoparone (SCO, 50 μmol/L), control or RAC1 overexpression adenovirus (10 moi) for 48 h. A, Western blot of RAC1‐GTP, t‐RAC1, NOX2 and NOX4 (n = 3); B, Immunofluorescent staining of RAC1‐GTP (n = 3); C.ROS assay (n = 3). *P < 0.05 vs control group; ^#^ *P* < 0.05 vs Ang II group; ^†^ *P* < 0.05 vs Ang II + SCO group

According to the results in Figure 4, SCO (50 μmol/L) inhibited the Ang II‐induced ROS generation and the oxidative stress‐related proteins such as RAC1‐GTP and total RAC1, NOX2 and NOX4 in NRCMs. RAC1 overexpression with adenovirus could eliminate the above‐mentioned effects of SCO. Thus, we can hypothesize that SCO decreased Ang II‐induced oxidative stress and expression of NOX2 and NOX4 in cardiomyocytes by the inhibition of RAC1.

**FIGURE 5:**
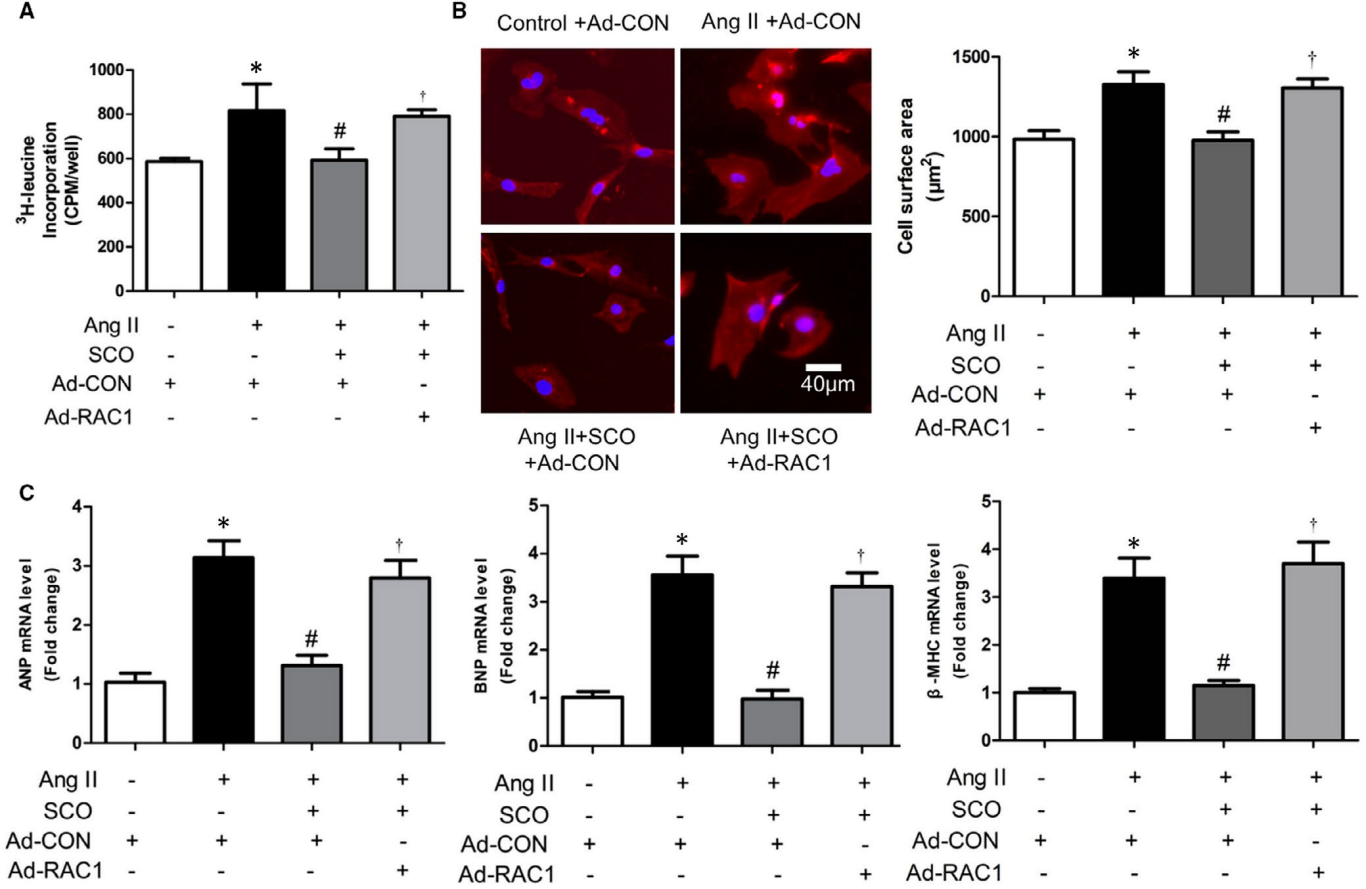
Scoparone inhibits Ang II‐induced cardiomyocyte hypertrophy by suppressing Rac1 mediated oxidative stress. NRCMs were treated with Ang II (0.1 μmol/L), scoparone (SCO, 50 μmol/L), control or RAC1 overexpression adenovirus (10 moi) for 48 h. A, ^3^H‐leucine incorporation assay (n = 4). B, Measure of cardiomyocytes surface area with immunofluorescence staining of cardiac troponin I (CTNI) (n = 8). C, Real‐time PCR analysis of ANP, BNP and β‐myosin heavy chain (β‐ MHC) mRNA level (n = 3).*P < 0.05; ^#^ *P* < 0.05 vs Ang II group; ^†^ *P* < 0.05 vs Ang II + SCO group

However, whether decreased RAC1 derived ROS was crucial for the anti‐hypertrophic effect of SCO remains to be assessed. Figure 5 showed that SCO (50 μmol/L) decreased Ang II‐induced cardiomyocyte protein synthesis, enlargement and foetal gene expression (ANP, BNP and β‐MHC). However RAC1 overexpression prevented SCO from the anti‐hypertrophic effect. It can be concluded that SCO inhibits Ang II‐induced cardiomyocyte hypertrophy by suppressing Rac1 mediated oxidative stress.

### 3.8 SCO inhibits Ang II‐induced cardiac fibroblasts differentiation and collagen synthesis by suppressing Rac1 mediated oxidative stress

**FIGURE 6:**
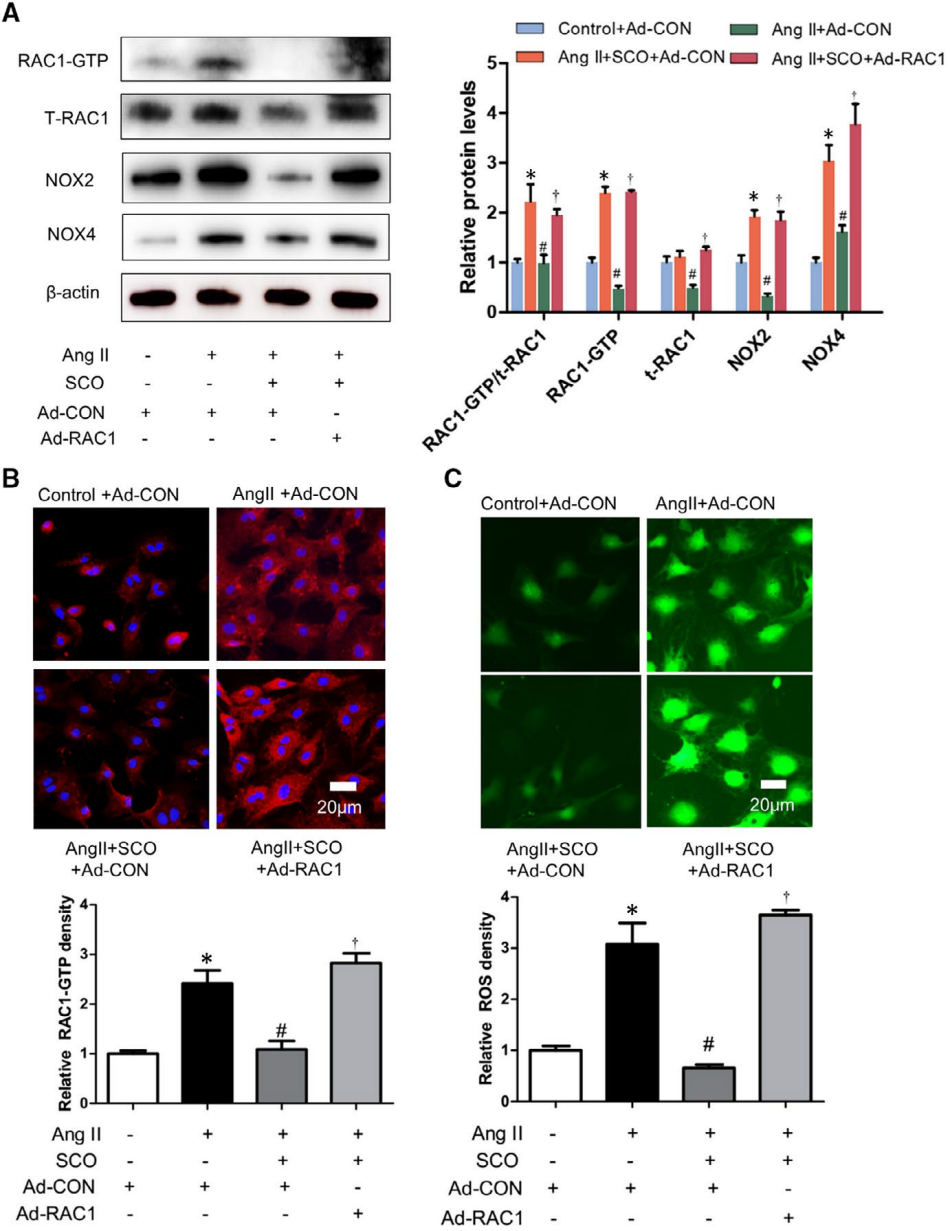
Scoparone inhibits Ang II‐induced cardiac fibroblasts oxidative stress by suppressing Rac1. NRCFs were treated with Ang II (1 μmol/L), scoparone (SCO, 50 μmol/L), control or RAC1 overexpression adenovirus (10 moi) for 48 h. A, Western blot of RAC1‐GTP, t‐RAC1, NOX2 and NOX4 (n = 3); B, immunofluorescent staining of p‐RAC1 (n = 3); C, ROS assay (n = 3). *P < 0.05 vs control group; ^#^ *P* < 0.05 vs Ang II group; ^†^ *P* < 0.05 vs Ang II + SCO group

**FIGURE 7:**
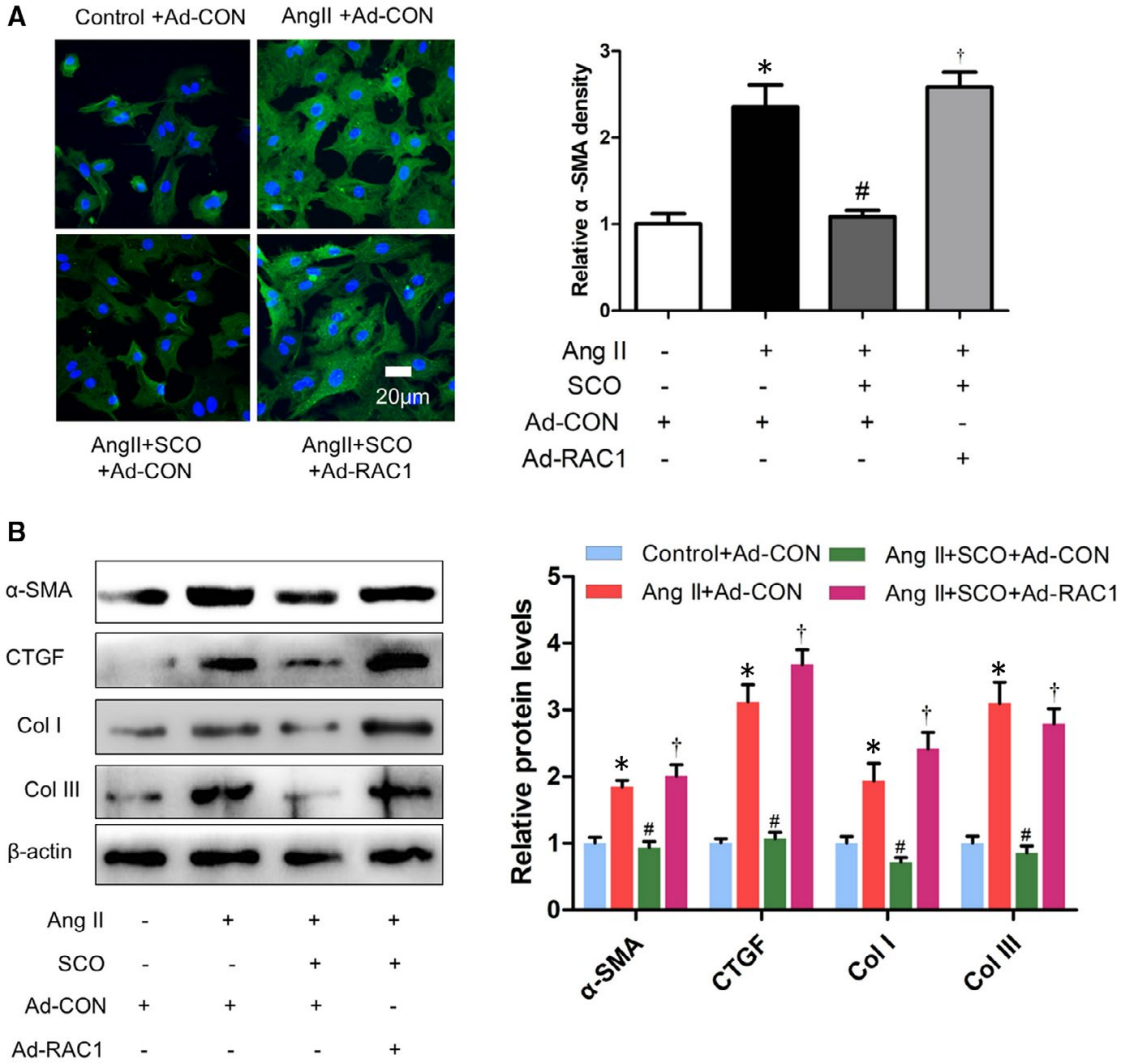
Scoparone inhibits Ang II‐induced cardiac fibroblasts differentiation and collagen synthesis by suppressing Rac1. NRCFs were treated with Ang II (1 μmol/L), scoparone (SCO, 50 μmol/L), control or RAC1 overexpression adenovirus (10 moi) for 48 h. A, Immunofluorescent staining of α‐SMA (n = 3). B, Western blot of α‐SMA, CTGF, collagen I and collagen III (n = 3). *P < 0.05 vs control group; ^#^ *P* < 0.05 vs Ang II group; ^†^ *P* < 0.05 vs Ang II + SCO group

Scoparone decreased Ang II‐induced oxidative stress and expression of NOX2 and NOX4 by inhibiting Rac1 protein levels in cardiac fibroblasts (Figure 6). As shown in Figure 7, SCO (50 μmol/L) blocked Ang II‐induced NRCF collagen synthesis and differentiation, and RAC1 overexpression prevented SCO from the above anti‐fibrotic effects. Overall, these findings indicate that SCO inhibits Ang II‐induced cardiac fibroblast differentiation and collagen synthesis by suppressing Rac1‐mediated oxidative stress.

## 4 DISCUSSION

Scoparone has been shown to exert pleiotropic effects including anti‐hypertension, vaso‐relaxation, anti‐angina, anti‐pancreatic fibrosis, pulmonary protection, hepatic protection, anti‐oxidative stress and anti‐inflammation.^15^, ^19^, ^25^, ^26^, ^27^, ^28^, ^29^, ^30^, ^31^ SCO also could also regulate osteoclastic cell differentiation, vascular smooth muscle cell proliferation, extracellular matrix accumulation in mesangial cells and hepatic stellate cell activation in vitro.^32^, ^33^, ^34^, ^35^ In our research, SCO has showed the ability to alleviate Ang II infusion‐induced cardiac hypertrophy and fibrosis (Figures 1 and 2). We found that SCO treatment significantly inhibited in vitro Ang II‐induced cardiomyocyte hypertrophy and cardiac fibroblast collagen synthesis and transformation into myofibroblasts (Figures S2 and S3). These results demonstrate the potential of SCO in anti‐pathological myocardial hypertrophy, which has not been reported before.

Pathological myocardial hypertrophy is closely related to Ang II‐induced oxidative stress,^36^, ^37^, ^38^ which could be mediated by RAC1, NOX2 and NOX4.^6^, ^8^, ^13^, ^14^, ^39^, ^40^, ^41^ In Figure S5, we proved that the protein levels of NOX2 and NOX4 could also be regulated by RAC1. This finding would help us deepen our understanding of the relationship between RAC1 and NOXs. In Figure 3, SCO showed strong anti‐oxidative ability in the hearts of Ang II‐infused mice. SCO could also inhibit the expression of RAC1‐GTP and total‐RAC1, NOX2 and NOX4 in Ang II‐infused mice hearts. Figures 3, 4 and 6 show that, both in vivo and in vitro, SCO could suppress RAC‐1 GTP and total‐RAC1 expression in cardiomyocytes and cardiac fibroblasts. Figures 4 and 6 show that overexpression of RAC1 eliminated the anti‐oxidative action of SCO in cardiomyocytes and cardiac fibroblasts. These data indicate that the anti‐oxidative effect of SCO was based on the blockage of the RAC1/NOXs axis. Moreover, SCO may also directly inhibit NOX2 and NOX4.

RAC1 is necessary for the development of myocardial hypertrophy. In Ang II‐stimulated mice, cardiomyocyte‐specific knockout of RAC1 resulted in decreased NADPH oxidase activity, cardiac hypertrophy and myocardial oxidative stress.^6^ Overexpression of a dominant‐negative mutant form of RAC1 (N17RAC1) suppresses phenylephrine‐induced cardiomyocyte oxidative stress and hypertrophy in vitro.^40^ Figure 5 shows that SCO could inhibit Ang II‐induced cardiac hypertrophy in vitro, and overexpression of RAC1 eliminated the above effect. Therefore, SCO may block Ang II‐induced cardiac hypertrophy by inhibiting RAC1‐mediated oxidative stress. It is well known that RAC‐GTP is also involved in cardiomyocytes hypertrophy by regulating mitogen‐activated protein kinases (MAPKs), apoptosis signal‐regulating kinase (ASK) 1 and a transcriptional factor, nuclear factor‐B (NF‐B).^41^, ^42^ Therefore, the anti‐hypertrophic effect of SCO based on the inhibition of RAC1 is not only by ROS regulation, but also via anti‐inflammation.

In T1DM mice, RAC1 deficiency reduced myocardial fibrosis and hypertrophy, resulting in improved myocardial function.^43^ In Ang II‐stimulated NRCFs, knockdown of RAC1 decreased the expression of CTGF.^8^ Figure 7 shows that SCO could inhibit Ang II‐induced cardiac fibroblast differentiation and collagen synthesis in vitro, and overexpression of RAC1 could eliminate the above effect. Hence, SCO blocks Ang II‐induced cardiac fibrosis by inhibiting RAC1 mediated oxidative stress.

In conclusion, we have demonstrated a novel function of SCO in the protective effect of Ang II‐induced myocardial hypertrophy both in vitro and in vivo, and the effect may be mediated by inhibition of RAC1‐dependent oxidative stress (Figure S6). Our study provides insight into the future treatment of myocardial hypertrophy through the application of SCO.

## CONFLICT OF INTEREST

None.

## AUTHOR CONTRIBUTIONS

**Linmao Lyu:** Data curation (lead); Funding acquisition (supporting); Software (lead); Writing‐original draft (lead). **jiazheng Chen:** Project administration (lead); Visualization (supporting). **Wei Wang:** Project administration (supporting); Visualization (supporting). **Tao Yan:** Project administration (supporting). **Jiamao Lin:** Writing‐review & editing (supporting). **Hongmei Gao:** Writing‐review & editing (supporting). **Hui Li:** Writing‐review & editing (supporting). **Ruijuan Lv:** Conceptualization (supporting); Writing original draft (lead). **Lijun Fang:** Conceptualization (equal); Funding acquisition (lead); Investigation (lead); Supervision (lead); Writing‐original draft (lead). **Yuguo Chen:** Conceptualization (equal); Funding acquisition (lead); Investigation (lead); Supervision (lead).

## Supporting information

Fig S1

Fig S2

Fig S3

Fig S4

Fig S5

Fig S6

Table S1

## Data Availability

The data will be made available after been required upon request from the corresponding author.
