# Supplementary figures and images for: Scoparone alleviates Ang II‐induced pathological myocardial hypertrophy in mice by inhibiting oxidative stress
Source: J Cell Mol Med. 2021 Feb 9;25(6):3136–48. doi: 10.1111/jcmm.16304 (PMC7957216; doi:10.1111/jcmm.16304)

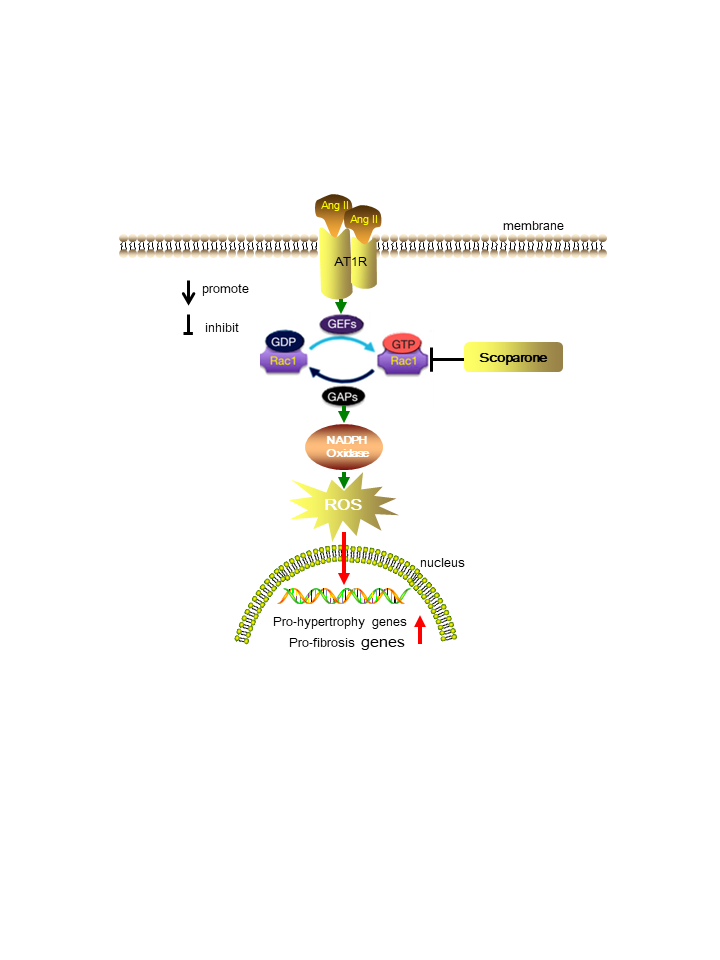

Supplement: Supplementary file 1 — Fig S1 [file JCMM-25-3136-s004.TIF]

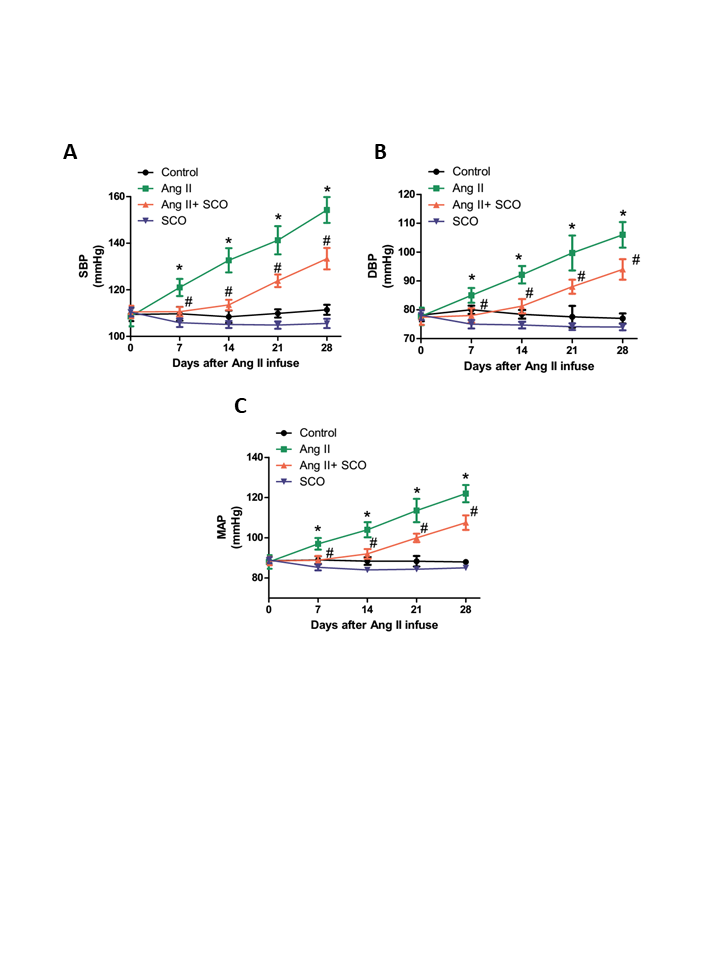

Supplement: Supplementary file 2 — Fig S2 [file JCMM-25-3136-s003.TIF]

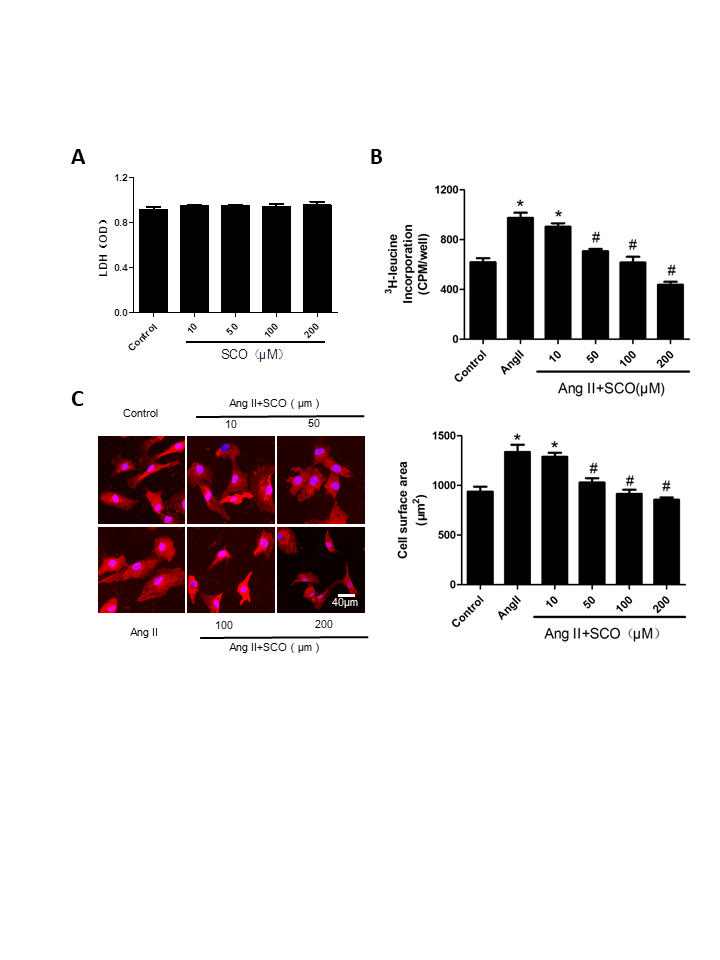

Supplement: Supplementary file 3 — Fig S3 [file JCMM-25-3136-s006.TIF]

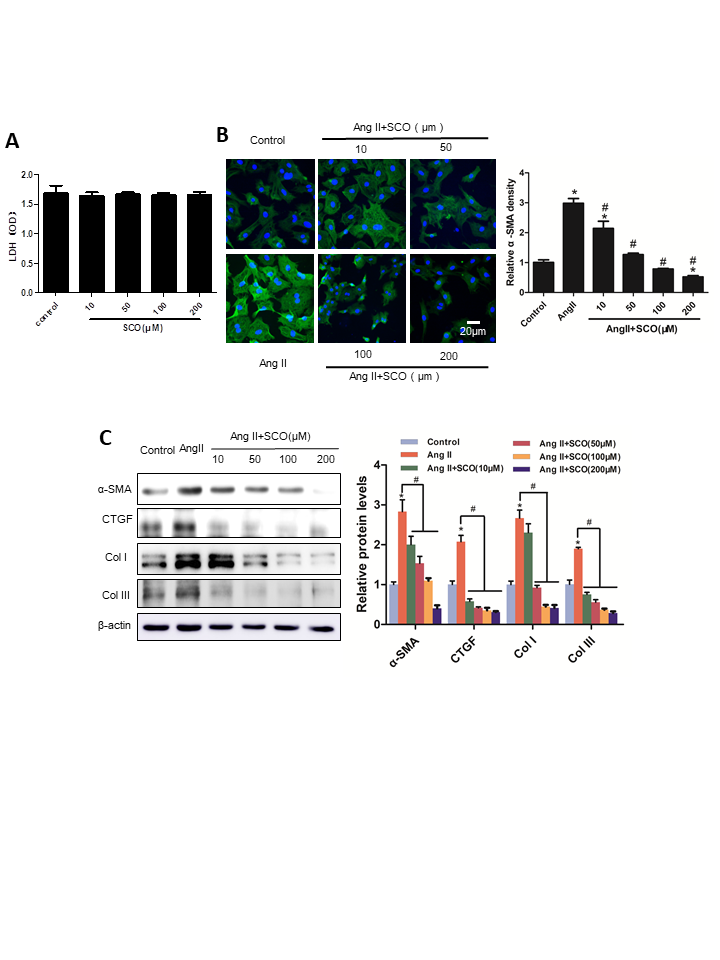

Supplement: Supplementary file 4 — Fig S4 [file JCMM-25-3136-s001.tif]

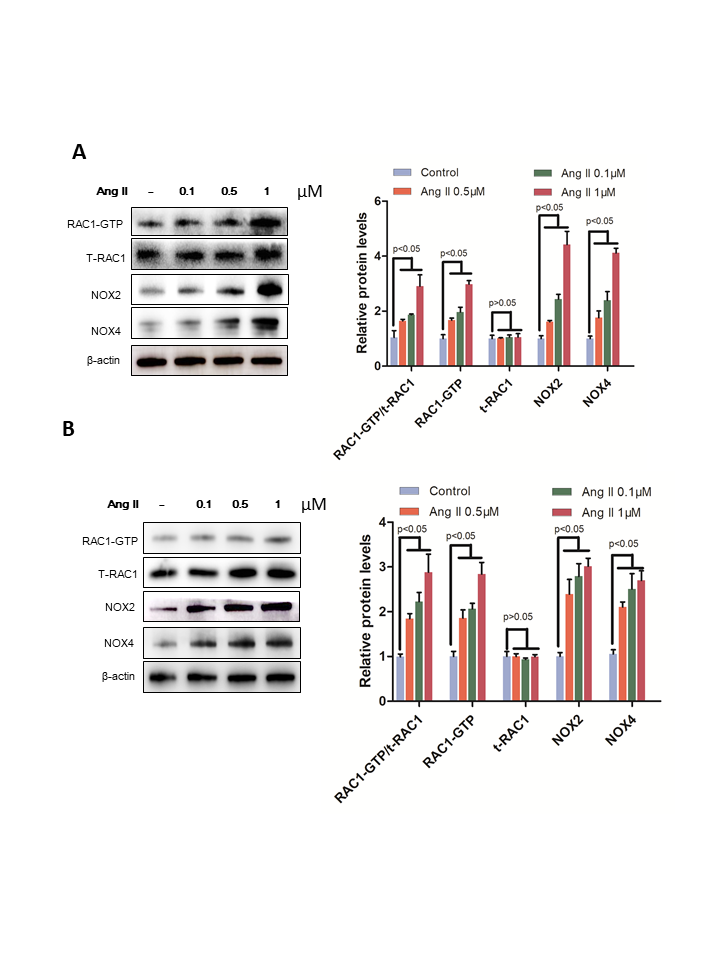

Supplement: Supplementary file 5 — Fig S5 [file JCMM-25-3136-s002.tif]

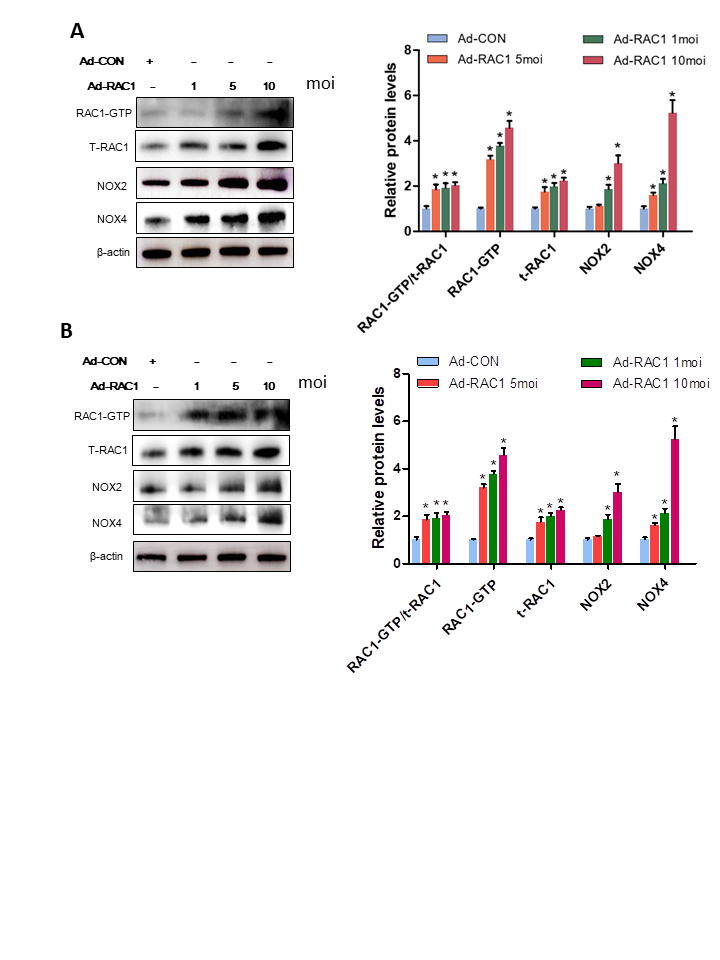

Supplement: Supplementary file 6 — Fig S6 [file JCMM-25-3136-s007.tif]

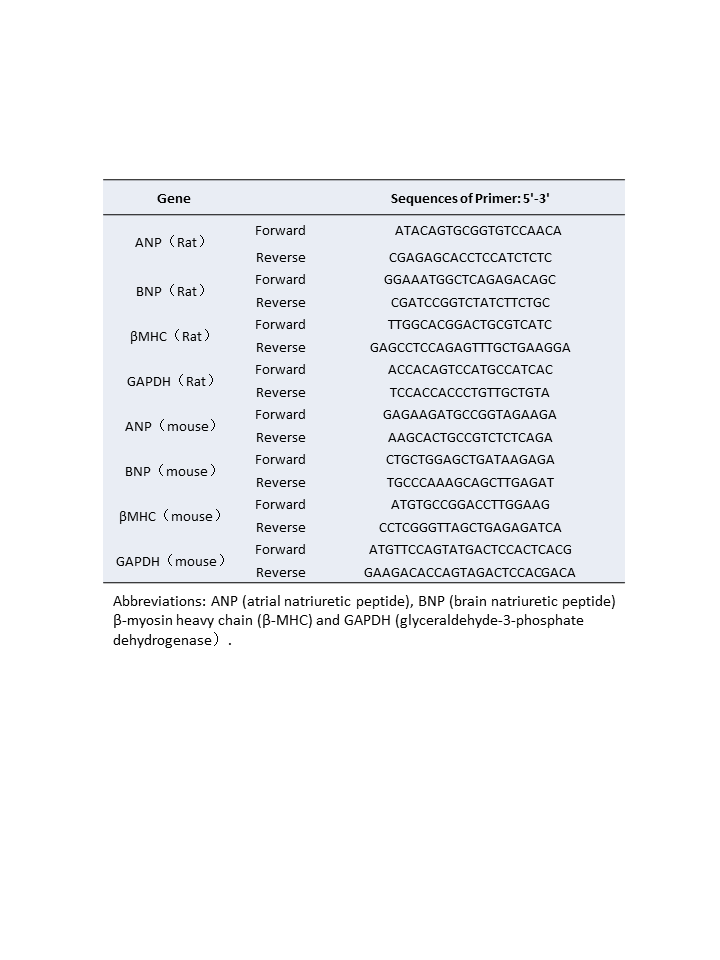

Supplement: Supplementary file 7 — Table S1 [file JCMM-25-3136-s005.TIF]
